# Educational attainment of childhood cancer survivors: A systematic review

**DOI:** 10.1002/cam4.2154

**Published:** 2019-04-21

**Authors:** Michal Molcho, Maureen D'Eath, Audrey Alforque Thomas, Linda Sharp

**Affiliations:** ^1^ Whitaker Institute, NUI Galway Galway Ireland; ^2^ School of Psychology NUI Galway Galway Ireland; ^3^ Office of Planning & Analysis UC Berkeley San Franscisco California; ^4^ Institute of Health & Society Newcastle University Newcastle upon Tyne United Kingdom

**Keywords:** cancer, childhood all, educational attainment, survivorship, systematic review

## Abstract

**Background:**

Advances in treatment mean that most children diagnosed with cancer during childhood survive. Therefore, it is increasingly important to examine the long‐term consequences of childhood cancer, including educational attainment. This systematic review investigated whether the educational attainment of childhood cancer survivors differ from the cancer‐free population.

**Design/methods:**

We searched seven databases for articles published from January 2005 to August 2018. We identified full papers in English, reporting primary data on academic attainment of adult survivors of childhood cancer, compared to a control group. Quality appraisal was conducted using the Newcastle‐Ottawa Scale.

**Results:**

Fourteen studies met the inclusion criteria. Nine papers included patients with various types of cancers, four focused on a single type of cancer, and one on patients who underwent stem cell transplantation.

Of the 14 papers, 2 studies were considered good quality, 10 were considered adequate quality, and 2 were considered poor quality. Four studies reported more favorable educational attainment among survivors while six did not report significant differences. Less favorable attainment was consistently reported for CNS survivors in four studies.

**Conclusion:**

The literature does not provide a clear pattern of the long‐term consequences of childhood cancer on education attainment. While this may suggest that there is no consistent difference between the education attainment of cancer survivors and controls, it may also be the result of limitations in the existing research. To better assess the education attainment of survivors, there is a need for high‐quality studies, with appropriate comparators, and standardized measures of education attainment across countries.

## 1 INTRODUCTION

Cancer is increasingly recognized as a chronic illness. The life trajectory of those diagnosed is no longer one of inexorable decline. Instead, around 66% of all patients and around 80% of childhood cancers patients now survive 5 years or longer.^1^, ^2^ This means that the population of childhood cancer survivors is rising; in the USA, for example, there are almost 400 000 childhood cancer survivors,^3^ and around 100 000 in Europe.^4^

With the rapid improvement in childhood cancer survival over the past three decades, increasing attention is being paid to the quality of survival. Studies have highlighted a range of difficulties experienced by childhood cancers survivors, including physical, social, and emotional problems.^5^, ^6^ It is estimated that, due to the toxicity of the available treatments, up to 90% of survivors of childhood cancer experience some late effects of treatment, and around one‐third have a severe, disabling, or life‐threatening chronic conditions.^3^, ^8^ Survivors have a 6‐11 times higher risk of death than the general population and, even 45 years after treatment, the risk is three‐times higher than expected.^9^, ^10^ In terms of morbidities, alongside pain, functional impairment, and impaired mental health, 35% of survivors have neurocognitive dysfunction,^3^ that can impact the academic performance of children with cancer and, potentially, result in poorer educational outcomes.

Education is a key predictor of future employment, income, and, in general, integration in society. Thus, educational attainment may be considered a key measure of the quality of long‐term survivorship. As well as the impact that treatment may have on cognition,^11^, ^12^ the education of survivors may also be adversely affected by missing time in school due to treatment, thus falling behind on schoolwork.^18^ Some studies estimate that 50% of children with cancer attend school in the first month after start of treatment. This increases to 70% of children in month 4 after start of treatment,^19^ but absenteeism in children with brain tumors is higher than for those with other cancers.^20^, ^21^ Irregular school attendance has been reported to last years after the end of treatment,^21^ adding to the educational difficulties experienced by survivors.^5^, ^22^, ^23^ However, while the cognitive effects of treatment have been extensively investigated,^3^ and interventions for addressing educational difficulties have been introduced in some settings,^24^, ^25^ to date, a systematic review of the international evidence on educational outcomes among survivors has not been published. This paper reports the results of a systematic review that investigated the long‐term effects of cancer on the educational attainment of childhood cancer survivors compared to children without cancer.

## 2 METHODS

### 2.1 Search strategy

**Figure 1:**
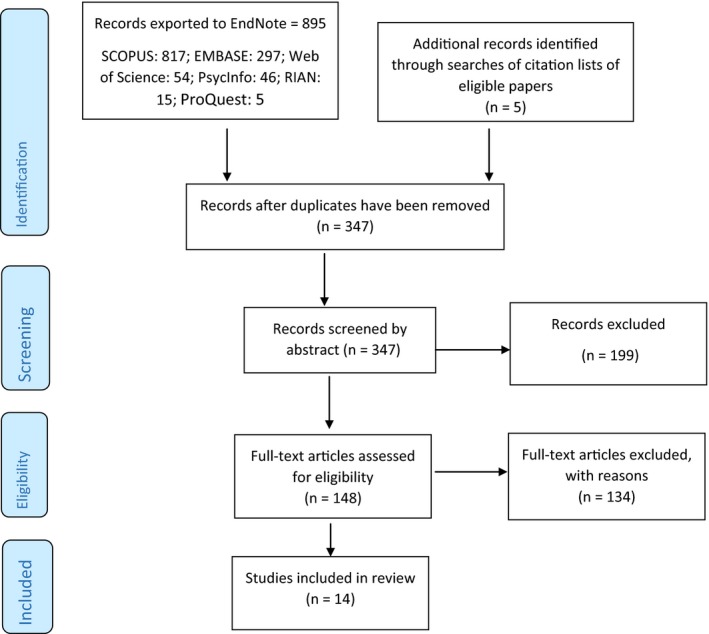
PRISMA flow diagram of selection process

We adhered to the Preferred Reporting Items for Systematic Reviews and Meta‐Analyses guidelines in conducting this review and preparing the manuscript.^26^ We searched seven databases (SCOPUS, Web of Science, EBSCO, EMBASE, ProQuest, RIAN, and the Applied Social Sciences Index and Abstracts), as well as citation lists of eligible papers, to identify studies reporting educational attainment of childhood cancer survivors published during 1 January 2005‐3 August 2018. The search combined terms (both MeSH headings and keywords) for the population of interest and those relating to educational attainment, including: childhood cancer, cancer, childhood, survivor, outcome, psychosocial, edu*, attain*, achieve*. Figure 1 shows the number of papers identified, screened, and included.

### 2.2 Eligibility criteria

For the purposes of this review, a childhood cancer was defined as a cancer diagnosed in someone up to 18 years of age. This allowed us to focus on cancers that occurred at the time where most children/adolescents would still be in an education setting. Educational attainment was defined as graduating primary school and/or secondary school, or attending or graduating college/university.

To be eligible, papers had to: be full papers; have a quantitative design; report primary data; include participants that were adults (18 years of age or over) at the time of data collection and who were diagnosed with cancer (or treated with a treatment usually used for cancer) when they were younger than 18; report educational attainment; include a noncancer control group or external comparison population; be published in a peer‐review journal, in English; and be conducted in a high‐income country.^27^

### 2.3 Data extraction

Titles and abstracts were screened independently by at least two reviewers (MM, MDE, AAT, LS). Full text of abstracts that were  considered potentially eligible were obtained and assessed for eligibilaty by two reviewers; in the event of disagreemnt, the final decision was made by the lead author. Reasons for exclusion were recorded and papers could be ineligible for multiple reasons.

Data were extracted independently by three reviewers (MDE, MM, LS) from each eligible paper on: (a) study location; (b) study design; (c) sample size and source of cases and controls/comparator group; (d) age at diagnosis and at assessment; (e) year of diagnosis; (f) outcomes measured; (g) method of data collection from cases and controls/comparator group; and (h) findings.

### 2.4 Quality appraisal

Eligible studies were critically appraised independently by two reviewers (MM, LS) using a checklist based on the Newcastle‐Ottawa Scale (NOS).^28^ Disagreements were carefully discussed and consensus reached. Each paper was assessed on eight domains. Each domain was scored 0 where the domain was missing, 0.5 where the domain was partially addressed, and 1 (and for one domain, comparability of cases and controls/comparator population, 2) where the domain was fully addressed in the paper. The eight criteria were as follows: (a) Case definition: 1 if cases were well described; (b) Representativeness: 1 if the study was population based; (c) Selection of controls: 1 if included controls (rather than comparison to national statistics); (d) Definition of controls: 1 if clear, detailed information was provided; (e) Comparability of controls: 2 if individually matched; 1 if “selected” from other surveys and matched to cases; (f) Assessment of educational attainment: 1 if assessment was based on administrative records (rather than self‐report); (g) Ascertainment of educational attainment is the same in cases and controls: 1 if yes; (h) Nonresponse/loss to follow‐up: 1 if figures reported. We considered score of ≥8 as good quality, 5‐7 adequate quality and ≤4 poor quality.

### 2.5 Statistical analysis

The study designs, methods and populations, and the outcomes assessed were heterogeneous, hence, no formal statistical attempt was made to combine the findings in a meta‐analysis.

## 3 RESULTS

### 3.1 Study selection

Following exclusion of duplicates, the titles and abstracts of 347 citations were screened. Of these, 148 full‐text papers were obtained and read; 14 were eligible for inclusion in the review (Figure 1).^26^ Reasons for exclusion were: educational attainment not reported (n = 135), population at diagnosis or at assessment was out of the eligible age range (n = 59), reviews and commentaries (n = 65); no cancer‐free controls (n = 26); qualitative study design (n = 12); abstracts or conference proceedings (n = 8); not reporting primary data (n = 6); not peer reviewed (n = 6); data not collected from survivors (n = 6); not in English (n = 5); cancers not reported separate from other diseases (n = 5).

### 3.2 Characteristics of eligible studies

Eleven studies were from Europe, two were from the USA, and one from Turkey (Table 1). Two studies had a prospective design and involved linkage of national cancer registration, education, and population data^29^, ^30^; the remainder were retrospective and were based on self‐report of educational attainment. The two linkage studies identified the comparable population without cancer; six studies compared cancer survivors' outcomes with data from individuals who participated in various surveys^31^, ^32^; two compared results for survivors with national data^37^, ^38^; and five recruited, and collected data from, cancer‐free control groups (siblings, classmates, or samples of the general population).^36^, ^39^, ^40^ The number of childhood cancer cases included in the studies ranged from 51 to 2213. Ten studies included multiple types of cancer; two studies focused only on leukemia^37^, ^40^ and a further two, which included multiple cancer types, reported subgroup analyses for leukemias and/or other blood cancers^29^, ^32^; one study^39^ included only CNS tumors, and a further three reported results for the subgroup with these tumors^24^, ^27^, ^36^; one study included only Differentiated Thyroid Carcinoma (DTC).^36^

**Table 1:** Description of the studies included in the review

	*Country*	*Study design^1^*	*Sample size*		*Source(s) of data*		*Cancers included*	*Outcome assessment*	
			*Cases*	*Comparators*	*Cases*	*Comparators*		*Cases*	*Comparator*
Boman et al (2009)^39^	Sweden	R	531	996	Registry	Population survey	CNS	Survey	Survey
Boman et al (2010)^29^	Sweden	P; linkage	1457	1 457 805	Registry	Population register	ALL, Lymphoma, CNS, Brain tumor, Other	Education Register	Education Register
Dieluweit et al (2011)^31^	Germany	R	820	850	Registry	Socio Economic Panel study (matched)	ALL, Lymphoma, CNS, Neuroblastoma, Renal tumor, Bone tumor, Soft tissue tumor. GCT, Other	Self‐completion questionnaire	Face‐to‐face interview
Dumas et al (2016)^32^	France	R	2066	Not reported	Hospital	Household survey	ALL, Lymphoma, Bone or soft tissue sarcoma, Nephroblastoma, Others	Self‐completion questionnaire	Not reported
Gerhardt et al (2007)^41^	USA	R	56	60	Hospital	Class mates	ALL, Lymphoma, Other non‐CNS tumor	Face‐to face interviews	Face‐to face interviews
Ghaderi et al (2015)	Norway	P; data linkage	2213	1 212 623	Registry	Population register	Mixed (not specified)	Population register	Population register
Jacola et al (2016)^40^	USA	R	691	259	Hospital	Siblings	ALL	Self‐completion questionnaire	Self‐completion questionnaire
Jóhannsdóttir et al (2010)^33^	Sweden, Denmark, Finland, Norway, Iceland	R	247	1814	Registry	Census study	AML, Wilms tumor, Astrocytoma	Self‐completion questionnaire	Self‐completion questionnaire
Kuehni et al (2012)^34^	Switzerland	R	961	5207	Registry	Population survey	ALL, Lymphoma, CNS, Neuroblastoma, Retinoblastoma, Renal tumor, Hepatic tumor, Bone tumors, Soft tissue sarcoma, GCT, Other	Self‐completion questionnaire	Face‐to face interview
Löf et al (2011)^35^	Sweden	R	51	2180	Hospital	Living Condition Survey	Treated with SCT	Self‐completion questionnaire	Self‐completion questionnaire
Nies et al (2017)^36^	The Netherlands	R	39	Peer: 30; Population comparison: 508	Registry	Peer controls, Population survey	DTC	Self‐completion questionnaire	Self‐completion questionnaire
Stam et al (2005)^42^	The Netherlands	R	353	508	Hospitals	Family doctors	ALL, Lymphoma, Solid tumors, Brain tumors	Self‐completion questionnaire	Self‐completion questionnaire
Yagci‐Kupeli et al (2013)^38^	Turkey	R	201	Population means	Hospital	National statistics office	HL, NHL, CNS Rhabdomyosarcoma, Wilms tumor, Langerhans cell, GCT, Other	Face‐to‐face interviews	Population means
Zynda et al (2012)^37^	Germany	R	1476	13 572	Registry	National statistics office	ALL	Self‐completion questionnaire	Population means

1 R = retrospective; P = prospective.

Ten studies reported both school‐ and third‐level educational attainment; usually this represented the highest level of education attained although the specific outcomes were different across studies. One study only reported school‐level education^41^ and one study only reported postschool education.^33^ In one study, the level of education was reported, but it was unclear whether these referred to school level alone or both school and postschool education.^36^ Only seven studies used the same methods to ascertain educational outcomes from the cancer cases and the comparison group.^29^, ^30^, ^35^, ^36^, ^40^, ^41^ In general the studies did not report the age of the survivors (and comparators) were when educational attainment was assessed.

### 3.3 Quality assessment

Two studies scored 9 out of a possible 9 and were considered good quality (Table 2).^29^, ^30^ Two studies scored 4 or less and were considered poor quality.^37^, ^38^ The remaining 10 studies were considered adequate quality. On average, studies scored well in terms of selection of controls/comparator population, but much more poorly on representativeness of cases, ascertainment of educational attainment, and comparability of ascertained of educational attainment in cases and controls/comparator population.

**Table 2:** Quality assessment of studies included in the review

	Case definition	Representativeness of cases	Selection of controls/comparison population	Definition of controls/comparison population	Comparability of controls/comparison population with cases*	Assessment of educational attainment	Comparability of ascertainment of educational attainment in cases and controls/comparison population	Nonresponse/lost to follow‐up	Quality rating
Boman et al (2009)^39^	1	0.5	1	0	1	0.5	0.5	1	5.5/9
Boman et al (2010)^29^	1	1	1	1	2	1	1	1	9/9
Dieluweit et al (2011)^31^	0.5	0.5	1	1	2	0.5	0	0.5	6/9
Dumas et al (2016)^32^	0.5	0.5	1	0.5	1	0.5	0	1	5/9
Gerhardt et al (2007)^41^	1	0	1	0	1	0.5	1	0.5	5/9
Ghaderi et al (2015)	1	1	1	1	2	1	1	1	9/9
Jacola et al (2016)^40^	0.5	0.5	1	0.5	2	0.5	1	1	7/9
Jóhannsdóttir et al (2010)^33^	1	1	1	1	0.5	0.5	0	0.5	5.5/9
Kuehni et al (2012)^34^	0.5	1	1	1	1	0.5	0	1	6/9
Löf et al (2011)^35^	0.5	0	1	1	1	0.5	0	0.5	4.5/9
Nies et al (2017)^36^	1	0.5	1	1	1	0	1	1	6.5/9
Stam et al (2005)^42^	1	0	1	1	2	0.5	1	1	7.5/9
Yagci‐Kupeli et al (2013)^38^	0.5	0	0.5	0	0.5	0	0	0	1.5/9
Zynda et al (2012)^37^	1	1	0.5	0	0.5	0.5	0	0	3.5/9

0, not addressed in paper; 0.5, partially addressed; 1 (or 2 for one criteria, indicated by *), fully addressed.

Summary of findings: Poor quality (0‐4): 2; adequate quality (5‐7), 10; good quality (8‐9), 2.

### 3.4 School‐level educational attainment: studies of multiple cancer types

Of the nine studies which included multiple cancer types, three reported significantly better school‐level outcomes in survivors than controls^31^, ^32^, ^38^ (Table 3). In contrast, in one study, significantly more survivors than controls completed only compulsory schooling (9% vs 5%; *P* < 0.001).^34^ The remaining studies either found modestly, but nonsignificantly, poorer educational attainment in survivors (n = 3)^30^, ^41^, ^42^ or no notable difference in educational outcomes between survivors and controls (n = 2).^29^, ^35^

**Table 3:** Main findings from the studies in the review, for school‐level and postschool education

	*Highest level of education: School*	*Risk estimate*	*Highest level of education: Postschool*	*Risk estimate*	*Adjustment factors*
Boman et al (2009)^39^	*Did not complete primary school*: survivors: 8.3%; controls 1.7% *Completed primary school*: survivors: 9.6%; controls: 4.4% *Completed secondary school*: survivors: 33.7%; controls: 22.4%	Not reported	*Entered third‐level education:* survivors: 16.9%; controls: 17.6% *Completed third‐level education:* survivors: 31.2%; controls: 53.9%	Not reported	Highest level of educational achieved (*P* < 0.001) was adjusted for sex and age
Boman et al (2010)^29^	*Completed basic education only:* All survivors: 10.8% (leukemia 9.7%; lymphoma 7.0%; CNS 15.5%; bone tumors 3.7%; other cancers 9.4%); controls 8.8% *Highest attained education*—*secondary*: All survivors: 54.6% (leukemia 57.9%; lymphoma 53.5%; CNS 55.0%; bone tumors 57.4%; other cancers 49.2%); controls: 54.4%	*Basic education only*: Hematological cancers: RR = 1.07 (0.79‐1.45) CNS tumors: RR = 1.80 (1.45‐2.23) Other cancers: RR = 1.05 (0.82‐1.36)	*Post‐secondary education*: All survivors: 34.7% (leukemia 32.5%; lymphoma 39.5%; CNS 29.5%; bone tumors 39.0%; other cancers 41.4%); controls: 36.8%.	*Post‐secondary*: Hematological cancers: RR = 0.92 (0.79‐1.07) CNS tumors: RR = 0.69 (0.58‐0.81) Other cancers: RR = 1.09 (0.97‐1.22)	RR controlled for sex and year of birth, residency, socioeconomic status and maternal country of birth
Dieluweit et al (2011)^31^	*Obtained university entrance qualifications (high school degree)*: survivors: 52.4%; controls: 38.3%; *P* < 0.001	Not reported	*Obtained third‐level degree*: survivors 24.7%; controls 17.0%; *P* < 0.001	OR = 0.93 (0.65‐1.33)	OR controlled for school education
Dumas et al (2016)^g^, ^32^	*Highest attained education* *All diagnoses* *< middle school/no diploma* Survivors: 11.4%; Expected 16.8% *Middle school* Survivors: 6.3%; Expected 6.8% *Vocational school* Survivors: 26.0%; Expected: 24.1 *High school* Survivors: 17.4%; Expected: 18.7% *Hodgkin's lymphoma* *< middle school/no diploma* Survivors: 9.1%; Expected 18.7% *Middle school* Survivors: 9.1%; Expected 7.4% *Vocational school* Survivors: 24.8%; Expected: 25.6% *High school* Survivors: 20.7%; Expected: 17.7% *Bone or soft tissue sarcoma* *< middle school/no diploma* Survivors: 7.4%; Expected 18.6% *Middle school* Survivors: 6.0%; Expected 7.1% *Vocational school* Survivors: 25.5%; Expected: 26.0% *High school* Survivors: 14.8%; Expected: 17.3% *CNS tumor* *< middle school/no diploma* Survivors: 40.6%; Expected 17.5% *Middle school* Survivors: 6.7%; Expected 7.0% *Vocational school* Survivors: 30.3%; Expected: 24.2% *High school* Survivors: 6.9%; Expected: 18.4% *Leukemia* *< middle school/no diploma* Survivors: 11.5%; Expected 12.2% *Middle school* Survivors: 16.6%; Expected 5.8% *Vocational school* Survivors: 13.4%; Expected: 19.4% *High school* Survivors: 33.1%; Expected: 22.2% *Other diagnoses* *< middle school/no diploma* Survivors: 8.4%; Expected 16.6% *Middle school* Survivors: 4.6%; Expected 6.8% *Vocational school* Survivors: 27.4%; Expected: 24.0% *High school* Survivors: 17.2%; Expected: 18.8%	O/E = 0.7 (0.6‐0.8) O/E = 0.9 (0.8‐1.1) O/E = 1.1 (1.0‐1.2) O/E = 0.9 (0.8‐1.0) O/E = 0.5 (0.2‐0.9) O/E = 1.2 (0.6‐2.2) O/E = 1.0 (0.7‐1.4) O/E = 1.2 (0.8‐1.8) O/E = 0.4 (0.3‐0.6) O/E = 0.8 (0.5‐1.3) O/E = 1.0 (0.8‐1.2) O/E = 0.9 (0.6‐1.1) O/E = 2.3 (1.8‐2.9) O/E = 1.0 (0.5‐1.8) O/E = 1.3 (1.0‐1.7) O/E = 0.4 (0.2‐0.7) O/E = 0.9 (0.6‐1.5) O/E = 2.9 (1.9‐4.2) O/E = 0.7 (0.4‐1.1) O/E = 1.5 (1.1‐2.0) O/E = 0.5 (0.4‐0.6) O/E = 0.7 (0.5‐0.9) O/E = 1.1 (1.0‐1.3) O/E = 1.3 (1.1‐1.4)	*College* *All diagnoses* Survivors: 38.9%; Expected: 33.5% *Hodgkin's lymphoma* Survivors: 36.4%; Expected: 30.7% *Bone or soft tissue sarcoma* Survivors: 46.2%; Expected: 30.9% *CNS tumors* Survivors: 15.4%; Expected: 32.8% *leukemia* Survivors: 25.5%; Expected: 40.3% *Other diagnoses* Survivors: 42.4%; Expected: 33.8%	O/E = 1.2 (1.1‐1.3) O/E = 1.2 (0.9‐1.6) O/E = 1.5 (1.3‐1.7) O/E = 0.5 (0.3‐0.7) O/E = 0.6 (0.5‐0.9) O/E = 1.3 (1.1‐1.4)	Expected numbers take account of age and sex distribution of survivors
Gerhardt et al (2007)^41^	*High school graduates*: survivors: 39% (22); controls 43% (22); ns	Not reported			None
Ghaderi et al (2015)	*Highest attained education—intermediate* a: All survivors 67% (727) (CNS 53% (142); CNS‐directed therapy 71% (274); other cancers 72% (41)); controls 70% (852 063)	Not reported	*Completed undergraduate education*: All survivors 31% (418) (CNS 20% (69); CNS‐directed therapy 32% (136); other cancers 38% (213)); controls 35% (430 018) *Completed graduate education*: All survivors 7% (135) (CNS 4% (18); CNS‐directed therapy 5% (27); other cancers 10% (90)); controls 9% (102 987)	Not reported	None
Jacola et al (2016)^40^	*Highest attained education:* *Grades 1‐12* f Survivors treated with CRT: 13%; Survivors not treated with CRT 6%: Controls 3% *Graduated high school* f Survivors treated with CRT: 17%; Survivors not treated with CRT 11%: Controls 9% *Post high school, some college* f Survivors treated with CRT: 27%; Survivors not treated with CRT 29%: Controls 26%	Not reported	*Graduated college* *Survivors treated with CRT* Survivors: 43%; Controls: 65%; *P*<=0.0001 *Survivors not treated with CRT* Survivors: 53%; Controls: 65%; *P* = 0.016	Not reported	None
Jóhannsdóttir et al (2010)^33^	—	—	Completed academic education^b^: survivors 32%; controls 28%	OR = 1.33 (0.95‐1.88)	OR controlled for age and gender
Kuehni et al (2012)^34^	*Completed only compulsory schooling*: All ages*—*survivors 8.7%; controls 5.2%; *P* < 0.001 Aged 27 and older*—*survivors 4.6%; controls 5.9%; *P* = 0.284	OR = 2.25 (1.65‐3.07)	*Completed compulsory education* c: all ages*—*survivors 36.1%; controls 24.1%; *P* < 0.001 *Obtained university degree*: all ages*—*survivors 7.3%; controls 11.0%; *P* < 0.001; aged 27 and older—survivors 11.3%; controls 14.5%; *P* = 0.083 *Vocational training*: survivors 47.9%; controls 59.6%; *P* < 0.001.	Completed upper secondary education or greater^a^: OR = 1.36 (1.12‐1.74) Obtained university degree^a^: OR = 0.75 (0.54‐1.05)	OR controlled for age, sex, migration background, place of living, and language region
Löf et al (2011)^35^	*Completed compulsory education only*: aged 19‐24*—*survivors 12.5% (3); controls 14.8%; ns; aged 25‐42*—*survivors 3.7% (1); controls 7.2%; ns *Upper secondary schooling*: aged 19‐24*—*survivors 70.8% (17); controls 56.9%; n.s; aged 25‐42*—*survivors 44.4% (12); controls 42.5%; n.s	Not reported	*Completed tertiary education*: aged 19‐24*—*survivors 16.6% (4); controls 28.4%; ns; aged 25‐42*—*survivors 51.8% (14); controls 49.8%; ns	Not reported	None
Nies et al (2017)^36^	Low level h survivors: 18%; peer controls: 0% general population controls: 28% Medium level: survivors: 39%; peer controls: 40% general population controls: 48% High Level: survivors: 44%; peer controls: 60% general population controls: 19% Peer controls *P* < 0.05 Comparison *P* < 0.001	Not reported			None
Stam et al (2005)^42^	*Completed low level of education only* d: survivors 33.6%; controls 29.4% ns *Highest attained education—middle level* d: survivors 50.3%; controls 50.6% ns	Not reported	*Attained high level of education*: survivors 16.1%; controls 20.0%; ns	Not reported	None
Yagci‐Kupeli et al (2013)^38^	*Primary school only*: survivors 21.5% (43); controls 45.0%; *P* < 0.001 *Highest attained*—*high school*: survivors 55.5% (111); controls 29.9%; *P* < 0.001	Not reported	*Attended university*: survivors 23.0% (47); controls 11.1%; *P* < 0.001	Not reported	None
Zynda et al (2012)^37^	*Highest level of education‐ school‐leaving certificate* e *:* Survivors^h^: 15.4%; controls: 27.7% *Highest level of education—*intermediate *school‐leaving certificate* e: Survivors^h^: 33.4%; controls: 33.5% *Highest level of education—high school diploma* e: Survivors^h^: 51.2%; controls: 38.8%	Not reported			None

ns, not significant.

a Includes basic upper secondary education, final year upper secondary education, and postschool nontertiary education.

b Defined as 4 y or more at a university of similar educational institution.

c Additional schooling, usually during ages 19‐27, leading to higher degrees/managerial jobs in specific professions.

d Low: primary education, technical, and vocational training, lower and middle general secondary education; middle: middle vocational education, higher general secondary education, preuniversity education.

e Level attained or currently strived for.

f Percentages estimated by review authors from chart in appendix of Jacola et al; results for all survivors combined not reported in paper.

g Percentages calculated by review authors from figures given in paper.

h Level of education completed (ie, whether school or postschool) is not specified in the paper.

### 3.5 Postschool educational attainment: studies of multiple cancer types

Three of the nine studies which included multiple cancer types and reported postschool educational attainment found that survivors had significantly better postschool outcomes than controls.^31^, ^32^, ^38^ In contrast, in one study, a significantly smaller proportion of survivors than controls obtained a university degree (7% vs 11%; *P* < 0.001), but this was no longer significant when the analysis was limited to those aged 27 and older at time of assessment of educational attainment.^34^ The remaining five studies found no significant difference in postschool educational attainment among survivors and controls.^29^, ^30^, ^33^, ^35^, ^42^

### 3.6 Studies reporting individual cancers

All four studies that reported on CNS tumors found evidence of poorer educational attainment in survivors than cancer‐free controls.^29^, ^30^, ^32^, ^39^ Findings from the four studies that reported on hematological cancers were inconsistent.^29^, ^32^, ^37^, ^40^ Jacola et al^40^ found lower college graduation rates among ALL survivors than controls, and Dumas et al^32^ reported that significantly more leukemia survivors than controls completed only middle school or high school and fewer than expected were college graduates. In contrast, Zynda et al^37^ reported that more leukemia survivors than controls attained a high school diploma while fewer attained only a secondary school graduation. Boman et al^29^ found no difference in completion of either only basic education, or third‐level education, in leukemia or lymphoma survivors than controls.

In the single study which considered patients with bone tumors,^29^ there was little difference between than controls in undertaking postschool education. In the single study which reported findings for bone or soft tissue sarcoma survivors,^32^ significantly fewer survivors than controls completed less than middle school while significantly more than expected were college graduates. In a single study that reported findings for DTC survivors,^36^ significantly fewer survivors obtained high level of education compared to peer controls, however, significantly more survivors obtained high‐level education compared to comparators.

### 3.7 Treatment

Two studies reported subgroup analyses by treatment receipt.^30^, ^40^ Jacola et al^40^ reported that more survivors who received cranial radiotherapy (CRT) completed only grades 1‐12 of school than survivors who did not receive CRT or sibling controls. Similarly, fewer of those who received CRT graduated from college. Ghaderi et al^30^ considered the subgroup of survivors who received CNS‐directed therapy and found that this group—when diagnosed in 1975‐1994, under 5 years of age, or 10‐14 years—were significantly less likely to complete undergraduate education than controls.

## 4 DISCUSSION

This systematic review investigated whether there are differences in educational attainment between cancer survivors and controls, in an attempt to gain a deeper understanding of the long‐term effects of childhood cancer, across different cancers and educational systems. Despite the strict criteria that were applied for inclusion in the review, the studies still varied in design, outcome measures considered, cancers included, and the methods of reporting. The variation was exacerbated by the fact that they were based in different educational systems. Indeed, the extent of heterogeneity precluded any statistical combination of the findings. Moreover, only two of the studies were considered high quality^29^, ^30^ based on NOS ratings.

Of the 10 papers examining school completion as the highest level of education attainment, five reported a higher proportion of cancer survivors compared to controls completing school^31^, ^34^, ^37^, ^38^ while the other five reported no significant differences in school completion between survivors and controls.^29^, ^30^, ^35^, ^41^, ^42^ Of the nine studies that reported postschool education as the highest educational attainment, two reported a higher proportion of controls achieving postschool education compared to survivors,^30^, ^39^ two reported a higher proportion of survivors achieving postschool education compared to controls,^31^, ^38^ and five reported no difference between survivors and controls.^29^, ^33^, ^34^, ^35^ One paper reported level of education but without indicating what these levels represented, with contradictory findings when compared to controls and comparators.^36^ These contradictory findings limit the ability to draw clear conclusions on a pattern of educational attainment of childhood cancer survivors overall.

The studies that included only one cancer type or compared findings across cancer types focused mainly on CNS tumors and blood cancers. The four studies that reported results for CNS consistently reported that survivors had poorer academic attainment compared to controls,^29^, ^30^, ^32^, ^39^ which could be the result of exposure to cranial radiation,^8^, ^15^, ^16^ as was suggested by Ghaderi et al^30^ who examined CNS‐directed treatment for non‐CNS patients. The two studies that focused on leukemia both reported that survivors had favorable educational attainment compared to controls, although the findings were statistically significant in only one of them.^35^, ^37^ Favorable educational attainments for ALL survivors could be explained by the higher SES that has been associated with ALL in some settings^38^; SES is also positively associated with educational attainments in children^43^ in general, and in cancer survivors in particular.^20^ However, none of the studies adjust for SES in their analysis.

The evidence presented in this review does not point to consistently reduced educational attainment among childhood cancers in general. This is somewhat surprising given the known impact of treatment on cognition.^11^, ^12^ However, not all survivors would have had treatments that could have impacted on their cognitive abilities, and the studies reviewed generally did not provide detailed information on treatment. It is also possible that childhood cancer survivors received support to overcome the time spent out of school, or that they participated in intervention programs to ameliorate the adverse cognitive effects of the treatment.^44^, ^45^ Such interventions have been found to improve survivors' educational attainment^25^; however, information on access to, or receipt of, such support was not included in the articles included in the review.

The observed equivocal picture of educational attainment of childhood cancer survivors could also be partly explained by the heterogeneity in the available studies. Most studies scored poorly on how educational attainment was ascertained and comparability of this between cases and controls. These weaknesses were exacerbated by the fact that studies defined/categorized attainment in different ways, probably reflecting (at least in part) the differences in education systems, and the complexity of measuring educational attainment cross‐nationally. This highlights the need to develop standardized measures for assessing educational attainment, that is, measures that would allow for pooled analysis cross country for individual cancer types. However, given the differences in educational systems, reaching a consensus on how and when to assess survivors' attainment may be challenging. In the meantime, improved reporting of individual studies would be helpful. For example, Koch et al,^20^ provided a clear description of the Danish education system—including such information could, in itself, improve ability to compare findings across different education systems.

Few studies were clearly population based, indicating the need for larger, population based, longitudinal studies that will track cancer types and treatment, as well as attainment over time from primary, through secondary and third‐level education. Such information would allow researchers to better understand the differences in educational attainment of different groups of childhood cancer survivors and be better able to collect data on the use of interventions or supports related to education among survivors and assess their “real‐world” effectiveness.

## 5 LIMITATIONS

The limitations of this review can be divided into limitations related to the review process and limitations related to the evidence. While we have attempted to achieve a complete literature search, it is possible that we failed to identify some relevant papers. Our search was predominantly electronic, which may result in the exclusion of papers that could only be found in manual searches. Additionally, limiting the review to papers written in English will have excluded any otherwise eligible papers in other languages.

In terms of the evidence itself, the most substantial limitation is the heterogeneity of the studies; this limited our ability to compare and contrast the findings and to conduct a formal statistical combination of the results. The studies varied in the method of recruitment of cases and controls, most were retrospective and they differed in types of cancers and reporting on different cancers separately, and in the statistical analysis that was carried out. Additionally, of the 14 papers that were reviewed, only two were categorized as good quality on the NOS. While we were still able to draw some conclusions, the lack of high‐quality research in this area is a substantial limitation.

## 6 CONCLUSIONS

The evidence reviewed here paints a mixed and inconsistent picture of educational attainment among childhood cancer survivors. The evidence tentatively suggests that, in some cancers, mainly CNS, educational attainment of survivors is poorer than that for children without cancer; for other cancers, the evidence is less strong or inconsistent. These findings strongly demonstrate the need for high‐quality, population‐based studies, underpinned by a more coordinated and standardized data collection.

## CONFLICT OF INTEREST

No conflict of interest to declare.

## AUTHORS' CONTRIBUTION

MM writing: original draft, reviewing and editing, funding acquisition, MD writing: reviewing and editing, data curation, visualization. AAT writing: reviewing and editing, funding acquisition, project administration. LS writing: original draft, reviewing and editing, funding acquisition.
