# Supplementary material for: Educational attainment of childhood cancer survivors: A systematic review
Source: Cancer Med. 2019 Apr 21;8(6):3182–95. doi: 10.1002/cam4.2154 (PMC6558589; doi:10.1002/cam4.2154)
Supplement: Supplementary file 1 [file CAM4-8-3182-s001.docx]

**Table 1 Description of the studies included in the review**

|  | ***Country*** | ***Study design^$^*** | ***Sample size*** | | ***Source(s) of data*** |  | ***Period of diagnosis/ treatment: cases*** | ***Cancers included*** | ***Educational***  ***outcomes assessed*** | ***Outcome assessment*** | |
| --- | --- | --- | --- | --- | --- | --- | --- | --- | --- | --- | --- |
|  |  |  | ***Cases*** | ***Comparators*** | ***Cases*** | ***Comparators*** |  |  |  | ***Cases*** | ***Comparator*** |
| Boman et al., 2009 | Sweden | R | 531 | 996 | Registry | Population survey | 1982-2001 | CNS | - Did not complete primary school - Passed primary school - Passed upper secondary school - Entered university - Completed university | Survey | Survey |
| Boman et al., 2010 | Sweden | P; linkage | 1,457 | 1,457,805 | Registry | Population register | 1976-2002 | Mixed | - Basic education (<9 y) - Secondary education - Post-secondary education (>14 y) | Education Register | Education Register |
| Dieluweit et al., 2011 | Germany | R | 820 | 850 | Registry | Socio Economic Panel study (matched) | Up to 2003^£^ | Mixed | - University entry level qualification   College or university degree | Self-completion questionnaire | Face-to-Face interview |
| Dumas et al., 2016 | France | R | 2,066 | Not reported | Hospital | Household survey | 1948-2000 | Mixed | - <middle school/no diploma - Middle school - Vocational school - High school - College | Self-completion questionnaire | Not reported |
| Gerhardt et al., 2007 | USA | R | 56 | 60 | Hospital | Class mates | Not reported | Mixed | - High school graduates - Attending pot-school education | Face-to face interviews | Face-to face interviews |
| Ghaderi et al., 2015 | Norway | P; data linkage | 2,213 | 1,212,623 | Registry | Population register | 1965-1985 | Mixed | - Intermediate education - Undergraduate education - Graduate education | Population register | Population register |
| Jacola et al., 2016 | USA | R | 691 | 259 | Hospital | Siblings | 1970-1999 | ALL | - Grades 1-12 (< high school) - Graduated high school - Post-high school - Graduate college | Self-completion questionnaire | Self-completion questionnaire |
| Jóhannsdóttir et al., 2010 | Sweden, Denmark, Finland, Norway, Iceland | R | 247 | 1,814 | Registry | Census study | 1985-2001 | Mixed | - Academic education | Self-completion questionnaire | Self-completion questionnaire |
| Kuehni et al., 2012 | Switzerland | R | 961 | 5,207 | Registry | Population survey | 1976-2003 | Mixed | - Compulsory education - Upper secondary education - Vocational training - University degree | Self-completion questionnaire | Face-to face interview |
| Löf et al., 2011 | Sweden | R | 51 | 2,180 | Hospital | Living Condition Survey | 1978-2001 | Treated with SCT | - Compulsory education - Upper secondary education - Tertiary education | Self-completion questionnaire | Self-completion questionnaire |
| Nies et al., 2017 | The Netherlands | R | 39 | 30 peer controls;  508 general population controls | Registry | Identified by cases (peers); identified by GPs (general population) | 1970-2013 | DTC | - Level of education completed (low/medium/high) | Self-completion questionnaire | Self-completion questionnaire |
| Stam et al., 2005 | The Netherlands | R | 353 | 508 | Hospitals | Family doctors | Up to 1997^£^ | Mixed | - Low level of education - Middle level of education - High level of education | Self-completion questionnaire | Self-completion questionnaire |
| Yagci-Kupeli et al., 2013 | Turkey | R | 201 | Population means | Hospital | National statistics office | 1972-2009 | Mixed | - Primary school - High school - University | Face-to-face interviews | Population means |
| Zynda et al., 2012 | Germany | R | 1,476 | 13,572 | Registry | National statistics office | 1980-2004 | Leukemia | - Secondary school - Intermediate school-leaving cert - High school diploma | Self-completion questionnaire | Population means |

$ R=retrospective; P=prospective

*Register not population-based for those diagnosed older than 15

^£^ Earliest year of diagnosis/treatment unclear in paper
